# Maltodextrin-induced intestinal injury in a neonatal mouse model

**DOI:** 10.1242/dmm.044776

**Published:** 2020-08-27

**Authors:** Pratibha Singh, Lady Leidy Sanchez-Fernandez, David Ramiro-Cortijo, Pedro Ochoa-Allemant, George Perides, Yan Liu, Esli Medina-Morales, William Yakah, Steven D. Freedman, Camilia R. Martin

**Affiliations:** 1 Division of Gastroenterology, Beth Israel Deaconess Medical Center, Harvard Medical School, Boston, MA 02115, USA; 2 Division of Translational Research, Beth Israel Deaconess Medical Center, Harvard Medical School, Boston, MA 02115, USA; 3 Department of Neonatology, Beth Israel Deaconess Medical Center, Harvard Medical School, Boston, MA 02115, USA

**Keywords:** Intestinal injury, Maltodextrin, Necrotizing enterocolitis, Neonatal mouse model

## Abstract

Prematurity and enteral feedings are major risk factors for intestinal injury leading to necrotizing enterocolitis (NEC). An immature digestive system can lead to maldigestion of macronutrients and increased vulnerability to intestinal injury. The aim of this study was to test in neonatal mice the effect of maltodextrin, a complex carbohydrate, on the risk of intestinal injury. The goal was to develop a robust and highly reproducible murine model of intestinal injury that allows insight into the pathogenesis and therapeutic interventions of nutrient-driven intestinal injury. Five- to 6-day-old C57BL/6 mice were assigned to the following groups: dam fed (D); D+hypoxia+*Klebsiella pneumoniae*; maltodextrin-dominant human infant formula (M) only; M+hypoxia; and M+hypoxia+*K. pneumoniae.* The mice in all M groups were gavage fed five times a day for 4 days. Mice were exposed to hypoxia twice a day for 10 min prior to the first and last feedings, and *K. pneumoniae* was added to feedings as per group assignment. Mice in all M groups demonstrated reduced body weight, increased small intestinal dilatation and increased intestinal injury scores. Maltodextrin-dominant infant formula with hypoxia led to intestinal injury in neonatal mice accompanied by loss of villi, increased MUC2 production, altered expression of tight junction proteins, enhanced intestinal permeability, increased cell death and higher levels of intestinal inflammatory mediators. This robust and highly reproducible model allows for further interrogation of the effects of nutrients on pathogenic factors leading to intestinal injury and NEC in preterm infants.

This article has an associated First Person interview with the first author of the paper.

## Abstract (precis)

**Summary:** Using maltodextrin-dominant human infant formula feeding in combination with hypoxia, we developed a highly reproducible model of small intestinal injury in the neonatal mouse.

## INTRODUCTION

Necrotizing enterocolitis (NEC) is a life-threatening gastrointestinal disease in preterm infants, characterized by abdominal distension, feeding intolerance, inflammation, intestinal necrosis and bloody stools. It is one of the leading causes of morbidity and mortality in neonatal intensive care units (Zani and Pierro, 2015). The prevalence of NEC is between 5% and 12% in very-low-birth-weight infants (<1500 g), with a mortality rate of 25-50% (Hodzic et al., 2017; Isani et al., 2018; Rich and Dolgin, 2017; Shulhan et al., 2017). NEC is a complex and multifactorial disease, and three major risk factors are thought to contribute to the pathogenesis of this disease: prematurity, altered bacterial colonization and enteral feeding, with the risk of NEC greater in formula-fed versus breastmilk-fed infants (Neu and Walker, 2011; Niño et al., 2016). These multifactorial processes culminate in an unbalanced and unmitigated inflammatory response in the immature intestine, leading to intestinal injury (Nanthakumar et al., 2000).

Almost universally, enteral feedings are received prior to the onset of NEC (Kwok et al., 2019). Despite this fact, many of the murine models to characterize intestinal injury in the immature host concentrate on hypoxia and bacteria/lipopolysaccharide, with little attention to the interface between nutrition and nutrient processing and intestinal injury, except for using dam-fed mice as controls, confirming the protective effect of breast milk versus infant formula in this disease process. Preterm infants are born with developmental pancreatic insufficiency, with impaired protein, fat and carbohydrate digestion. Maldigestion of these macronutrients in the immature intestine may lead to impaired nutrient handling and intestinal injury (Howles et al., 1999; Sodhi et al., 2018). In preterm piglets, exposure to maltodextrin, a complex carbohydrate, is sufficient to induce an NEC-like intestinal injury (Buddington et al., 2018; Thymann et al., 2009). Based on these reports, we hypothesized that mice pups, which demonstrate pancreatic insufficiency during the early postnatal period, when fed a maltodextrin-containing human infant formula as the major source of carbohydrate, would also be at an increased risk of developing an NEC-like intestinal injury. The aim of the study was to test the effect of a maltodextrin-dominant infant formula on the development of intestinal injury and the additive impact of various stress factors such as hypoxia and *Klebsiella pneumoniae*. Ultimately, our goal was to develop a robust and highly reproducible murine model of intestinal injury that would allow insight into nutrient-driven pathogenesis and therapeutic interventions.

## RESULTS

### Mice fed maltodextrin-dominant infant formula exhibit reduced growth compared to dam-fed mice

**Fig. 1.**
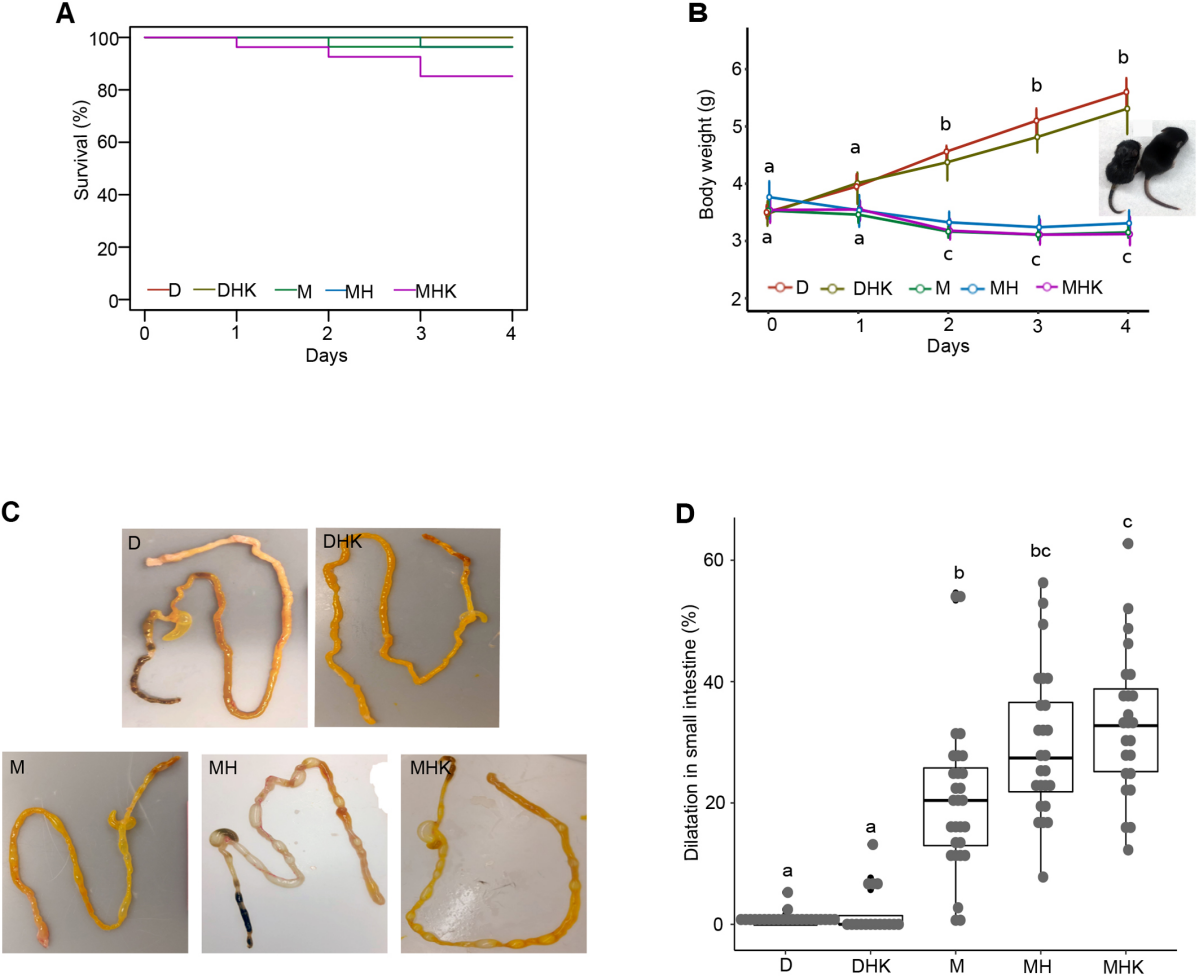
**Effects of maltodextrin-containing formula feeding on survival, body weight and gross intestinal appearance in neonatal mouse pups.** (A) Survival rate shown by Kaplan–Meier curves and tested by log-rank test. (B) Longitudinal body weights of mouse pups. The graph shows the median and IQR. Representative images at day 4 of mice in MH and D groups are shown on the left and right, respectively. (C) Gross images of representative samples of the small and large intestine from the different experimental groups. (D) Percentage dilatation in the small intestine presented as median and IQR. Labeled points without a common letter represent statistically significant differences, *P*<0.05. D, dam-fed mice, *n*=23; DHK, dam-fed mice administered *K. pneumoniae* (K) by oral gavage and subjected to hypoxia (H), *n*=16; M, mice fed a human infant formula containing maltodextrin as a major carbohydrate source alone, *n*=28; MH, mice fed maltodextrin-containing infant formula and subjected to hypoxia, *n*=25; MHK, mice fed maltodextrin-containing infant formula with *K. pneumoniae* and subjected to hypoxia, *n*=24.

The number of animals per group is depicted in Table 1. Litter size varied between six and 12 pups, and a total of 25 litters were used. In all cases, litters were randomly divided into experimental and control groups. Survival was monitored throughout the experiment. No spontaneous mortality was observed in the dam-fed alone (D) and D+hypoxia+*K. pneumoniae* (DHK) groups. There were no significant differences in survival rates across the groups (Fig. 1A; *P*=0.09). Survival at day 4 was 96% for maltodextrin alone (M) and M+hypoxia (MH) groups, and 85% for the M+hypoxia+*K. pneumoniae* (MHK) group, compared to 100% for groups D and DHK (Table 1, Fig. 1A).

**Table 1.**
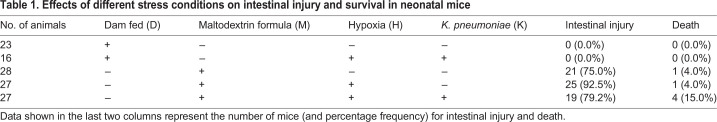
**Effects of different stress conditions on intestinal injury and survival in neonatal mice**

The median±interquartile range (IQR) body weight of group D increased continuously from 3.5±0.2 g at day 0 to 5.6±0.6 g at day 4 (Fig. 1B). Similarly, the body weight of the DHK group increased from 3.5±0.4 g at day 0 to 5.3±0.8 g at day 4. In contrast, all of the M groups did not gain body weight; rather they reduced their overall body weight over the course of the study (Fig. 1B). Body weight for the M group decreased from 3.5±0.3 g at day 0 to 3.2±0.2 g at day 4, for the MH group decreased from 3.8±0.7 g at day 0 to 3.3±0.4 g at day 4, and for the MHK group decreased from 3.5±0.4g at day 0 to 3.1±0.5 g at day 4. No statistically significant differences were observed between the two dam-fed groups (D and DHK groups) or between the three maltodextrin-fed groups (M, MH and MHK groups). On day 0 and day 1, the body weights were similar among all the groups (Fig. 1B); however, significant differences in body weights between the dam-fed groups and the maltodextrin-fed groups were noted at days 2, 3 and 4 (*P*<0.001).

### Maltodextrin-dominant infant formula induces intestinal injury in neonatal pups

During the 4 days of the protocol, maltodextrin-dominant infant formula**-**fed mice were more likely than dam-fed mice to have looser stools, demonstrate lethargy and develop abdominal distention. On gross examination, the gastrointestinal tract and other organs were normal in the D groups (D and DHK; Fig. 1C). In contrast, in all of the M groups, focal areas of dilatation were present, predominantly observed in the jejunum and ileum in a patchy distribution. In addition to the small intestine, the dilatation was also present in the large intestine. In some animals, the cecum was also dilated with fluid or air inside. Similar to the D groups, all other organs appeared normal on gross examination. The total length of the involved segments in the small bowel was quantified and presented as percentage dilatation of total small intestinal length (Fig. 1D). Median±IQR percentage dilatation was quantified as 0.0±0.0%, 0.0±1.4%, 20.4±12.8%, 27.4±14.7% and 32.7±13.6% in the D, DKH, M, MH and MHK groups, respectively. Compared to the D groups, the M groups showed statistically significant differences in small intestinal dilatation (*P*<0.001). Within the M groups, the small intestinal percentage dilatation in MHK was significantly greater than that in M, but not that in MH, while no difference was observed between the M and MH groups (Fig. 1D).

**Fig. 2.**
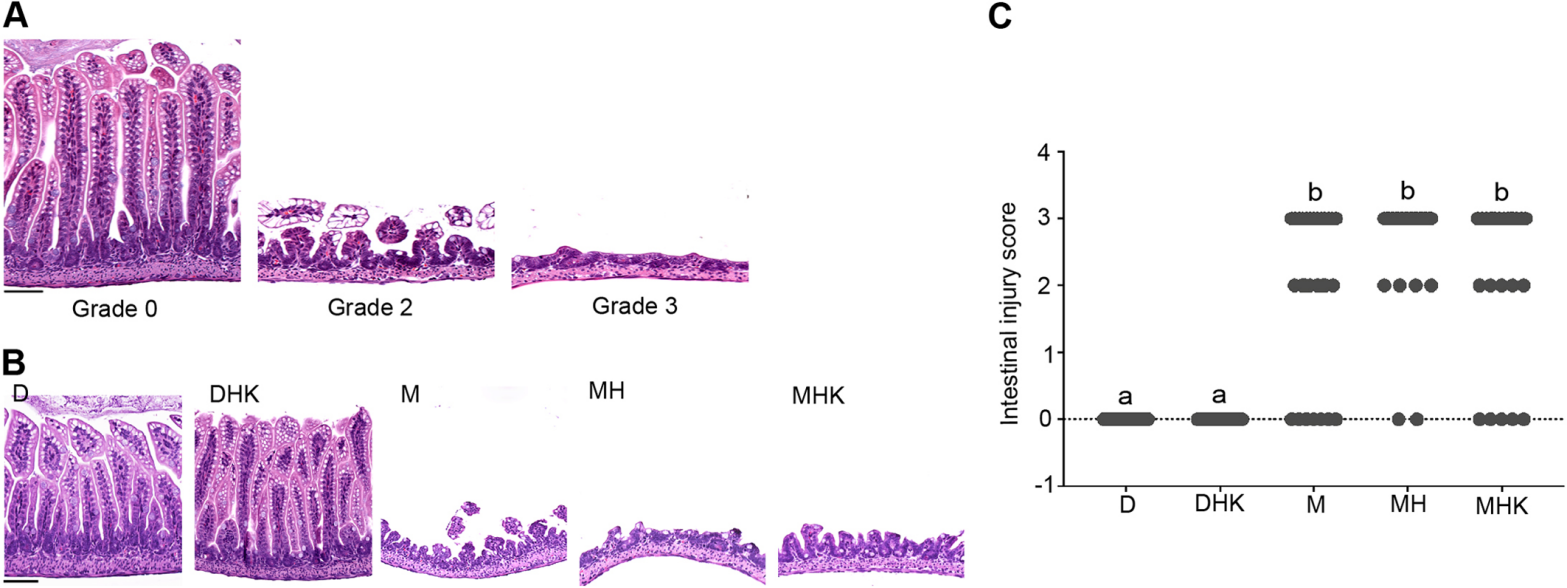
**Maltodextrin-containing formula feeding in neonatal mouse pups results in intestinal injury.** (A,B) H&E staining of distal ileum from experimental groups D, DHK, M, MH and MHK. Representative images (200×, scale bars: 50 μm) depicting the injury score of mouse distal ileum. Representative images with intestinal grading are shown in A: normal villus structure with intact crypt region (grade 0), partial villus loss resulting in shortening of villi (grade 2) and complete loss of villi (grade 3). Representative images from groups D, DHK, M, MH and MHK are shown in B. (C) Quantitative analysis of the injury score in the distal ileum shown as a scatter dot plot. Labeled points without a common letter represent statistically significant differences, *P*<0.05. D, dam-fed mice, *n*=23; DHK, dam-fed mice administered *K. pneumoniae* (K) by oral gavage and subjected to hypoxia (H), *n*=16; M, mice fed a human infant formula containing maltodextrin as a major carbohydrate source alone, *n*=28; MH, mice fed maltodextrin-containing infant formula and subjected to hypoxia, *n*=27; MHK, mice fed maltodextrin-containing infant formula with *K. pneumoniae* and subjected to hypoxia, *n*=24.

Hematoxylin-Eosin (H&E)-stained sections of the small intestine from the mouse pups of the D groups displayed normal intestinal morphology, intact architecture of intestinal epithelium, intact long villi and well-organized crypts at the base of villus (grade 0; Fig. 2A, left). Addition of bacteria and hypoxia to dam-fed mice did not result in any gross or histological abnormalities in group DHK. In all the M groups, there was either partial or complete loss of villi (grade 2 in Fig. 2A, middle, or grade 3 in Fig. 2A, right), which was limited to the areas that were dilated. In the unaffected areas (segments of the bowel without any distension), the villi appeared normal and the mucosa showed epithelial features similar to those of control group D. In addition to villus disruption, we infrequently observed separation of the lamina propria, which was always in combination with partial or complete villus loss, and thus was scored as grade 2 or 3. We did not observe areas of transmural necrosis (grade 4) in any experimental group. Representative images from different groups are shown in Fig. 2B. There was a significant increase in the injury score in all of the M groups (M, MH and MHK) compared to the D groups (Fig. 2C). Within the maltodextrin groups, 75% of the mice in the M group, 92.5% of the mice in the MH group and 79.2% of the mice in the MHK group showed an intestinal injury score ≥2.0 (*P*<0.001 for D versus each of the M groups). There was a significant positive correlation between intestinal injury and percentage dilatation (ρ=0.68, *P*<0.001).

### Maltodextrin-dominant formula results in increased intestinal cell death

**Fig. 3.**
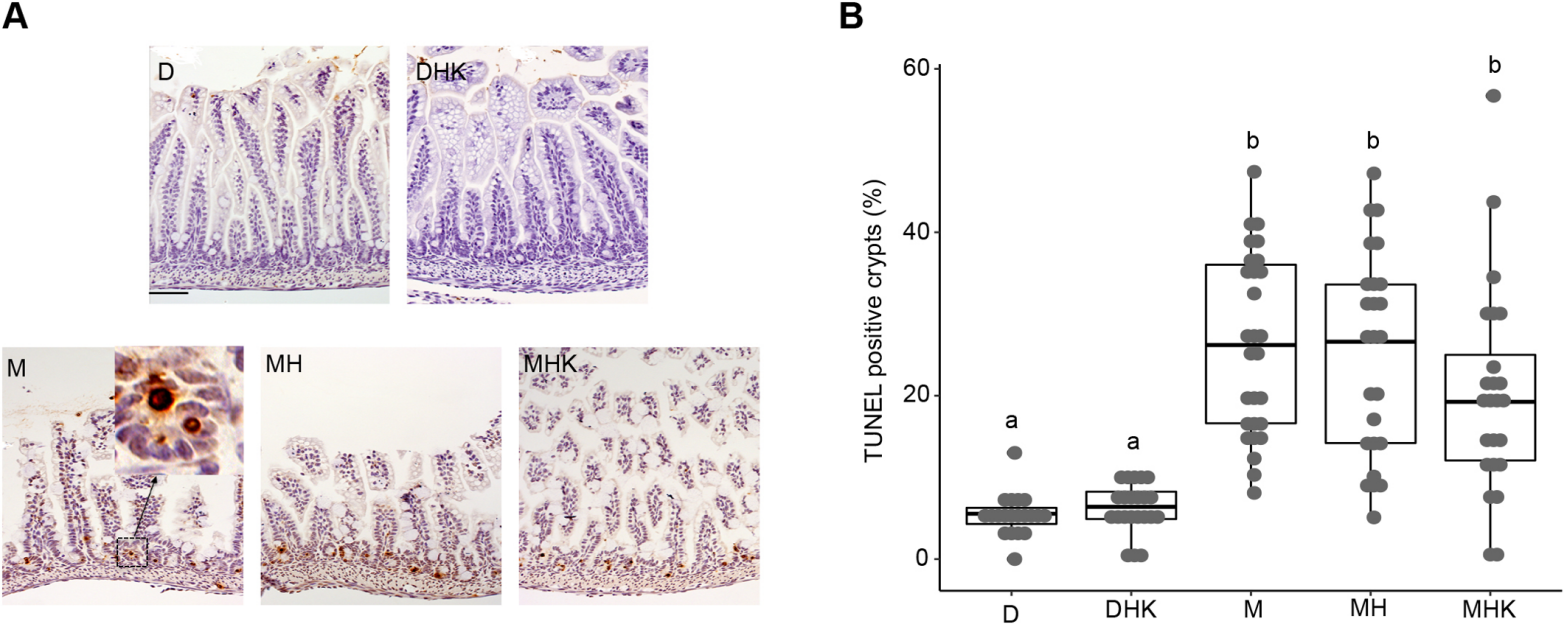
**Maltodextrin-containing formula feeding in neonatal mouse pups increases intestinal apoptosis.** (A) Representative images of distal ileum from different groups are shown for terminal deoxynucleotidyl transferase-mediated dUTP nick-end labeling (TUNEL) staining (200×, scale bar: 50 μm). The boxed region and higher-magnification image in the inset show specific TUNEL staining. (B) The percentages of TUNEL-positive crypts in distal ileum are shown as a box dot plot with median and IQR. Labeled points without a common letter represent statistically significant differences, *P*<0.05. D, dam-fed mice, *n*=23; DHK, dam-fed mice administered *K. pneumoniae* (K) by oral gavage and subjected to hypoxia (H), *n*=16; M, mice fed a human infant formula containing maltodextrin as a major carbohydrate source alone, *n*=28; MH, mice fed maltodextrin-containing infant formula and subjected to hypoxia, *n*=27; MHK, mice fed maltodextrin-containing infant formula with *K. pneumoniae* and subjected to hypoxia, *n*=24.

Apoptosis, determined by the percent of TUNEL-positive crypts, was significantly greater in the M groups compared to the D groups (Fig. 3A). Median±IQR percentage of TUNEL-positive crypts was 26.2±19.4%, 26.6±19.4% and 19.3±12.9% in the M, MH and MHK groups, respectively (Fig. 3B). In comparison, the percentage of TUNEL-positive crypts was 5.6±13.0% and 6.4±3.4% in the D and DHK groups, respectively. No differences were observed within the D and DHK groups, nor within the M groups, M, MH and MHK.

Cell proliferation, quantified by Ki67 (also known as MKI67) staining at the base of the crypts, was similar across all groups (Fig. S1A,B).

### Goblet cell density and MUC2 production in the small intestine in response to maltodextrin-dominant formula

**Fig. 4.**
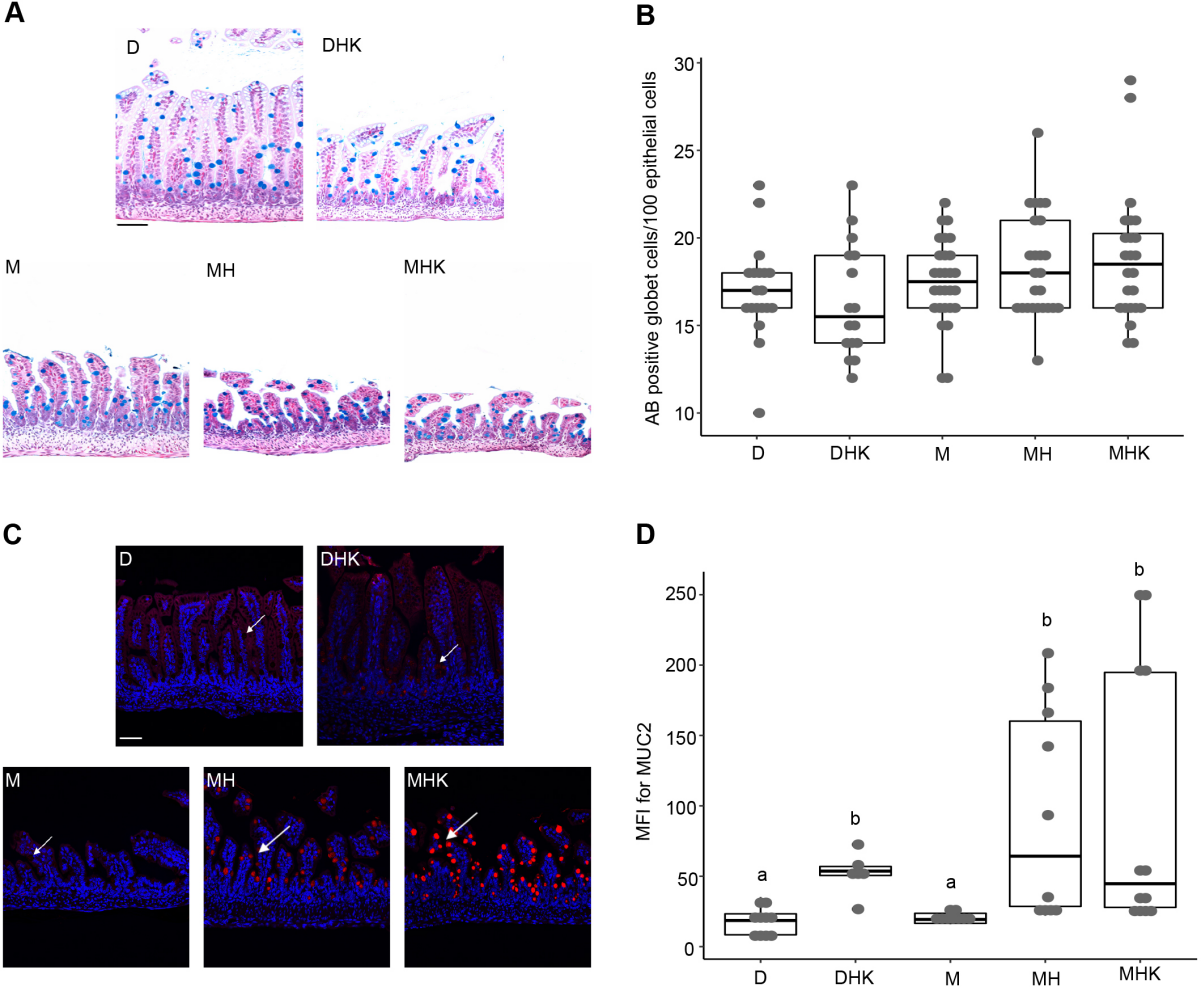
**Mice fed maltodextrin-containing formula in combination with hypoxia exhibit increased MUC2 production compared to dam-fed mice.** (A) Representative images (200×, scale bar: 50 μm) of AB-stained goblet cells are shown. (B) Box dot plot of AB-stained cells per 100 epithelial cells showing median and IQR for each experimental group. (C) Representative images of immunostained distal ileal segments with anti-MUC2 (red) detected by Cy3-conjugated secondary antibody. Nuclei were stained with DAPI (blue, images representative at 200×, scale bar: 50 μm). Arrows point to the MUC2-positive cells. (D) Quantification of mean fluorescence intensity (MFI) for MUC2-positive staining presented as a box dot plot showing median and IQR. Labeled points in panels B and D without a common letter represent statistically significant differences, *P*<0.05. D, dam-fed mice, *n*=23; DHK, dam-fed mice administered *K. pneumoniae* (K) by oral gavage and subjected to hypoxia (H), *n*=16; M, mice fed a human infant formula containing maltodextrin as a major carbohydrate source alone, *n*=28; MH, mice fed maltodextrin-containing infant formula and subjected to hypoxia, *n*=27; MHK, mice fed maltodextrin-containing infant formula with *K. pneumoniae* and subjected to hypoxia, *n*=24.

Ileal samples were stained with Alcian Blue (AB) to detect goblet cells (Fig. 4A). AB-positive cells were counted per 100 villus epithelial cells by an investigator blinded to the sample identity. There were no differences in the number of goblet cells across all the groups (Fig. 4B). Goblet cells are functionally characterized by the cytosolic accumulation of mucin and the expression of MUC2. The mean fluorescence intensity of MUC2 expression levels was significantly higher in the DHK (*P*=0.01), MH (*P*<0.01) and MHK (*P*<0.01) groups than in the dam-fed control group D (Fig. 4C,D). No differences were observed between the D alone and M alone groups, and between the MH and MHK groups (*P*=0.9 for both comparisons).

### Maltodextrin-dominant formula alters the expression of tight junction proteins

**Fig. 5.**
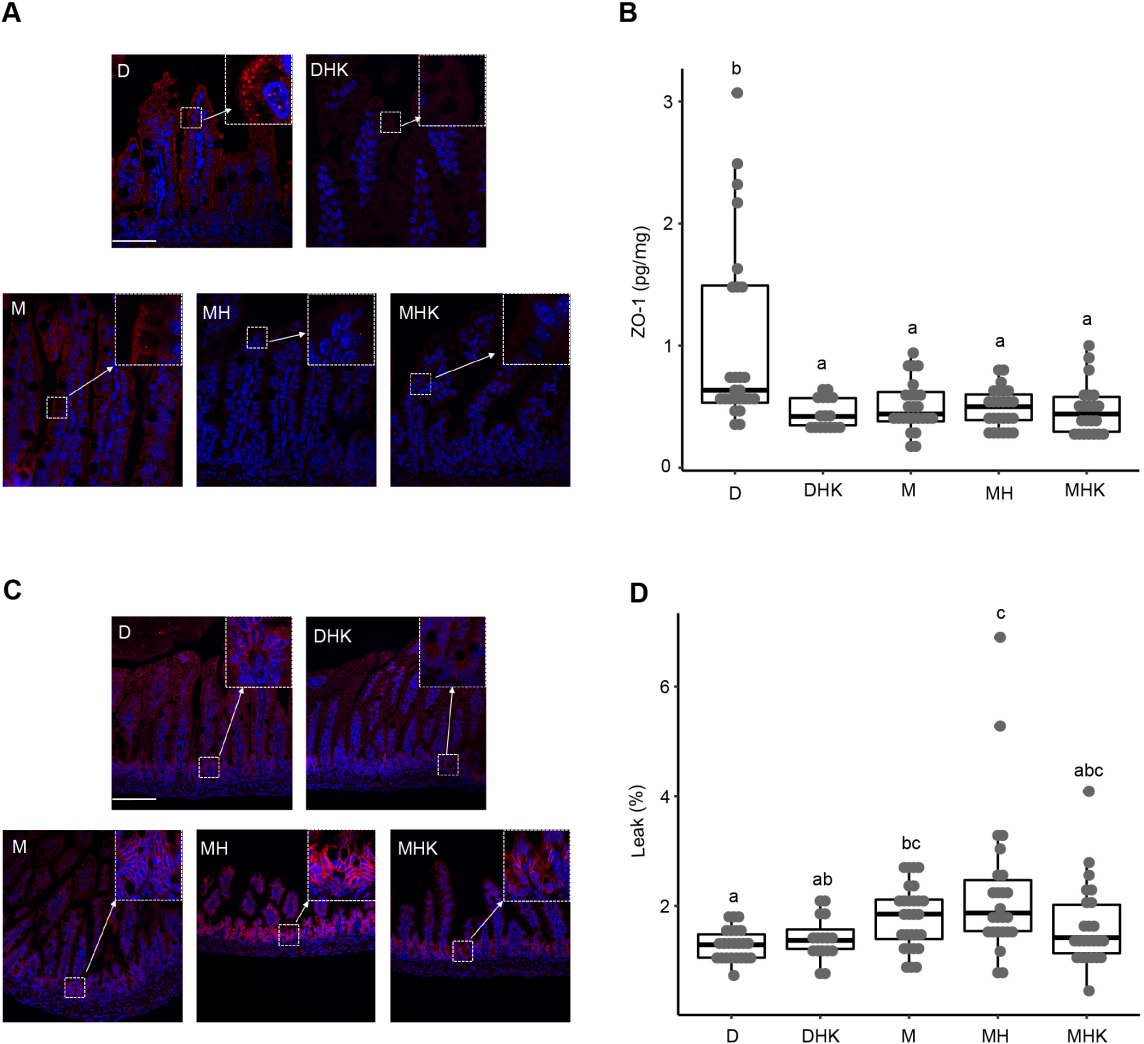
**Maltodextrin-containing infant formula feeding results in altered intestinal tight junction protein and barrier function.** (A) Representative images (400×, scale bar: 50 μm) of immunofluorescence staining of ZO-1 proteins in the distal ileum. Tissue sections were immunostained with anti-ZO-1 (red) and detected by the Cy3-conjugated secondary antibody. Nuclei were stained with DAPI (blue). The ZO-1 signal is indicated by arrows. Boxed regions correspond to higher-magnification images in the insets. (B) ZO-1 levels quantified by ELISA presented as a box dot plot showing median and IQR. (C) Representative images (200×, scale bar: 50 μm) of distal ileum sections immunostained with anti-claudin-3 (red) and detected by Cy3-conjugated secondary antibody. Nuclei were stained with DAPI (blue). The claudin-3 signal is indicated by arrows. Boxed regions correspond to higher-magnification images in the insets. (D) Percentage intestinal leak shown as a box dot plot with median and IQR. Labeled points in panels B and D without a common letter represent statistically significant differences, *P*<0.05. D, dam-fed mice, *n*=23; DHK, dam-fed mice administered *K. pneumoniae* (K) by oral gavage and subjected to hypoxia (H), *n*=16; M, mice fed a human infant formula containing maltodextrin as a major carbohydrate source alone, *n*=28; MH, mice fed maltodextrin-containing infant formula and subjected to hypoxia, *n*=27; MHK, mice fed maltodextrin-containing infant formula with *K. pneumoniae* and subjected to hypoxia, *n*=24.

Immunostaining for ZO-1 (also known as TJP1) (Fig. 5A), confirmed by enzyme-linked immunosorbent assay (ELISA) quantification (Fig. 5B), was decreased in all experimental groups compared to dam-fed control group D. The median±IQR protein concentrations of ZO-1 per μg total protein were 0.6±1.0pg/μg protein for group D, 0.4±0.2 pg/μg protein for DHK, 0.4±0.2 pg/μg protein for group M, 0.5±0.2 pg/μg protein for group MH, and 0.4±0.3 pg/μg protein for group MHK. Immunofluorescent microscopy showed increased immunostaining signal intensity for claudin-3 in the MH and MHK groups compared to group D (Fig. 5C). No differences in expression levels of claudin-3 between groups D, DHK and M were visible.

### Maltodextrin-dominant formula increases intestinal permeability

No significant differences in intestinal permeability, presented as percentage leak, were observed between the D groups, D and DHK (1.3±0.4% and 1.9±0.7%, respectively; *P*=0.5) (Fig. 5D). Compared to dam-fed control group D, intestinal permeability was significantly increased in groups M and MH (*P*=0.02 and *P*=0.005, respectively). No statistically significant differences were observed within the M groups.

### Maltodextrin-dominant formula induces early increases in intestinal inflammatory cytokine levels

Increased levels of inflammatory cytokines have been implicated in the pathogenesis of intestinal injury. Therefore, we investigated various cytokine profiles (Th1 and Th2) in intestinal homogenates from neonatal mice in our model of intestinal injury on day 4, at the end of the experimental protocol. The levels of Th1 cytokines such as IL-1β (*P*=0.01), KC/GRO (also known as CXCL1) (*P*<0.01) and TNF-α (also known as TNF) (*P*<0.01), as well as those of Th2 cytokines such as IL-4 (*P*<0.01) and IL-6 (*P*<0.01), were significantly different between the groups (Fig. S2). Compared to group D, the levels of IL-1β and TNF-α were significantly lower in group M (*P*<0.01) and group MH (*P*<0.01), respectively. TNF-α levels were also significantly lower in the MH and MHK groups compared to group DKH (*P*=0.02). IL-4 levels were significantly lower in groups M, MH and MHK, compared to group D (*P*<0.01), and in group MHK compared to group DHK (*P*<0.01). In contrast, the levels of KC/GRO were significantly higher in all three maltodextrin-fed groups compared to group DHK, and IL-6 levels were significantly higher in the MH group compared to the D (*P*<0.01) and DHK groups (*P*=0.02).

**Fig. 6.**
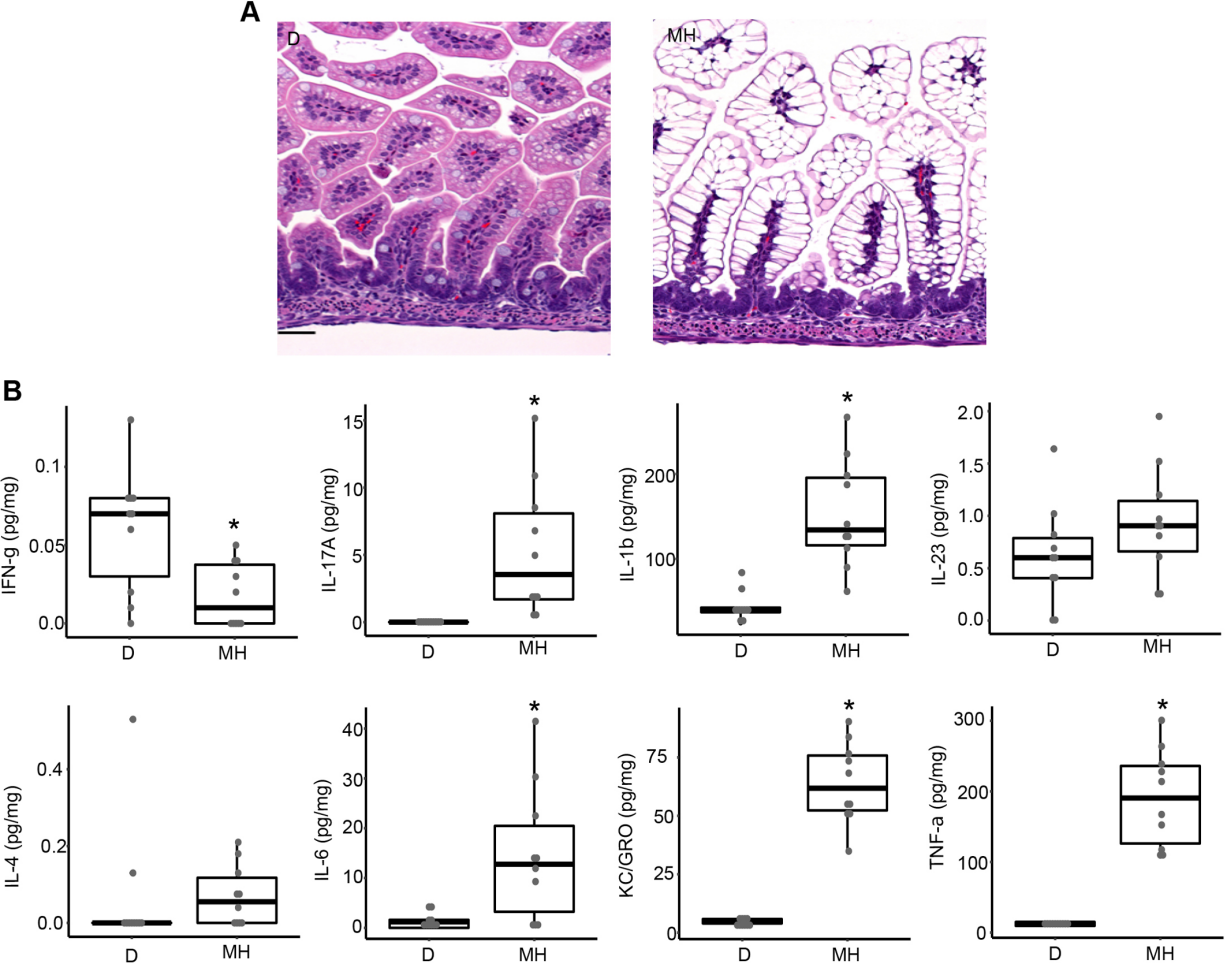
**Mice fed maltodextrin-containing formula exhibit increased cytokine production in intestinal tissues at 24 h compared to dam-fed mice.** (A) Small intestinal morphology at 24 h in mice fed maltodextrin-containing infant formula with hypoxia (right), compared to the control group, D (left) (representative images of H&E staining at 200×, scale bar: 50 μm). (B) Cytokine (IFN-γ, IL-17A, IL-1β, IL-23, IL-4, IL-6, KC/GRO and TNF-α) levels in intestinal tissue lysates at 24 h in the D and MH groups. Data are presented as box dot plots showing median and IQR. *P<0.05. Evaluation was limited to the D and MH groups as these two groups represented the extremes in observed intestinal injury. D, dam-fed mice, *n*=10; MH, mice fed maltodextrin-containing infant formula with hypoxia, *n*=10.

To evaluate the presence of inflammation prior to the development of morphological changes, and potential attenuation by day 4, we examined the histology of the distal ileum and intestinal tissue cytokines on day 1 in groups D and MH. This evaluation was limited to the D and MH groups, as these two groups demonstrated the largest differential in intestinal injury. On day 1, the intestinal segments from group MH showed vacuolated villi in the small intestine (Fig. 6A). In contrast, group D did not show vacuolated villi. Furthermore, no disruption of villi was observed in group MH compared to group D. Despite no differences in the gross morphology, many of the measured cytokines were significantly different between groups D and MH. The levels of IL-17A (*P*<0.001), IL-1β (*P*<0.001), IL-6 (*P*=0.02), KC/GRO (*P*<0.001) and TNF-α (*P*<0.001) were all significantly higher in group MH compared to group D (Fig. 6B). In contrast, IFN-γ levels were significantly lower in group MH compared to D (*P*=0.01). No differences in IL-23 (also known as IL23A) and IL-4 levels were found between groups D and MH.

Table S1 summarizes the temporal differences in cytokine levels from day 1 to day 4 of the protocol between the control group D and the MH group. On day 1, as described above, group MH had statistically significant increased IL-17A, IL-1β, IL-6, KC/GRO and TNF-α levels relative to group D; however, by day 4, the differences in IL-17A, IL-1β and KC/GRO had resolved. The TNF-α levels in the MH compared to D groups were lower at day 4 versus day 1, where previously there was no difference. In contrast to a resolution or lowering of cytokine responses, there was a persistent increase in IL-6 levels in the MH group relative to the D group (Table S1).

### Maltodextrin-dominant infant formula induces intestinal injury in a dose-dependent manner

**Fig. 7.**
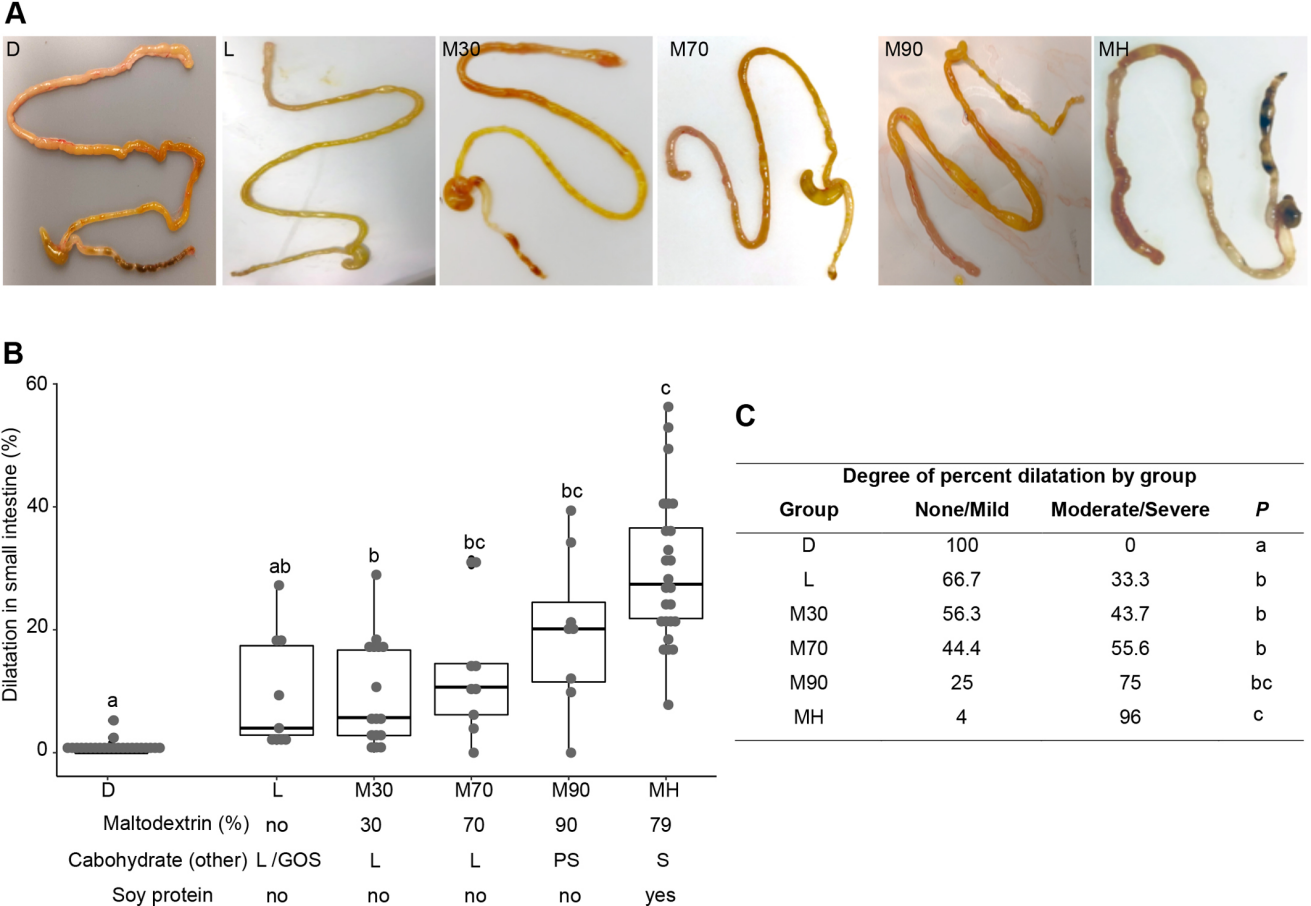
**Maltodextrin induces intestinal injury in a dose-dependent manner.** (A) Representative images of gross morphological changes in the small and large intestines from groups D, L, M30, M70, M90 and MH. (B) Percentage dilatation in the small intestine presented as a box dot plot showing median and IQR. Maltodextrin (%), other carbohydrate sources (GOS, galactooligosaccharide; L, lactose; PS, potato starch; S, sucrose), and the presence of soy (no/yes) is shown for the different formulas; the absence of soy indicates whey as the source of protein. (C) Categorization of percentage dilatation as none/mild or moderate/severe. The proportion of mice in each group (%) by diet are shown. ‘None/mild’, intestinal dilatation less than the median percentage for the total population (median, 10.4%); ‘moderate/severe’, intestinal dilatation greater than the median percentage for the total population. Labeled points without a common letter represent statistically significant differences, *P*<0.05. D, dam-fed mice, *n*=23; L, mice fed lactose containing human infant formula and subjected to hypoxia, *n*=9; M30, mice fed 30% maltodextrin-containing infant formula and subjected to hypoxia, *n*=16; M70, mice fed 70% maltodextrin-containing infant formula and subjected to hypoxia, *n*=9; M90, mice fed 90% maltodextrin-containing infant formula and subjected to hypoxia, *n*=8; MH, mice fed 79% maltodextrin-containing infant formula and subjected to hypoxia, *n*=25.

When the nutrient composition is compared between lactose-dominant and maltodextrin-dominant formulas, aside from the carbohydrate source, the other difference is the protein source, whey versus soy (Table 2, Table S2). The other components represented only slight variations. Thus, to further interrogate the role of maltodextrin present in human infant formula in intestinal injury in our model, we tested different human infant formulas across a gradient of maltodextrin content and protein source. In addition to the control dam-fed group (D) and our study group with maximal injury, maltodextrin-dominant (79% maltodextrin+21% sucrose) soy formula (M) mice were fed formula with 94% lactose and whey (L), 30% maltodextrin (M30)+70% lactose and whey, 70% maltodextrin (M70)+30% lactose and whey, and 90% maltodextrin (M90)+10% potato starch and whey (Table 2, Table S2). Relative to dam-fed group D, all other infant formula-fed groups showed changes in gross morphology (Fig. 7A). Median±IQR percentage dilatation was quantified as 0.0±0.0%, 4.0±14.6%, 5.7±13.9%, 10.7±8.3%, 20.1±13.0% and 27.4±14.7% in the D, L, M30, M70, M90 and MH groups, respectively. The groups fed lactose-containing formula did not show significant changes in percentage dilatation compared to D. In contrast, the maltodextrin-fed groups showed significant difference in percentage dilatation compared to group D (*P*<0.05). Group MH showed significantly higher percentage dilatation compared to groups L (*P*=0.03) and M30 (*P*=0.002), while no significant difference was observed between groups MH, M70 and M90 (MH vs M70; *P*=0.12, MH vs M70; *P*=0.94; Fig. 7B). The proportions of mice with percentage dilatation in the moderate/severe category versus the none/mild category increased with increasing percentage maltodextrin in the infant formula (Fig. 7C). None of the mice in the dam-fed group had moderate/severe intestinal dilatation. In contrast, all of the maltodextrin-containing formula groups had some degree of moderate/severe injury, the extent of which correlated with maltodextrin content. The proportion of mice with moderate/severe intestinal dilatation was 33.3%, 43.7%, 55.6%, 75% and 96% for the L, M30, M70, M90 and MH groups, respectively (Fig. 7C). The lack of significance difference between the high-maltodextrin whey formula (M90) and the high-maltodextrin soy formula (MH) suggests that maltodextrin versus protein source is the primary driver; however, a potential interaction between maltodextrin and the protein source could not be fully excluded.

**Table 2.**
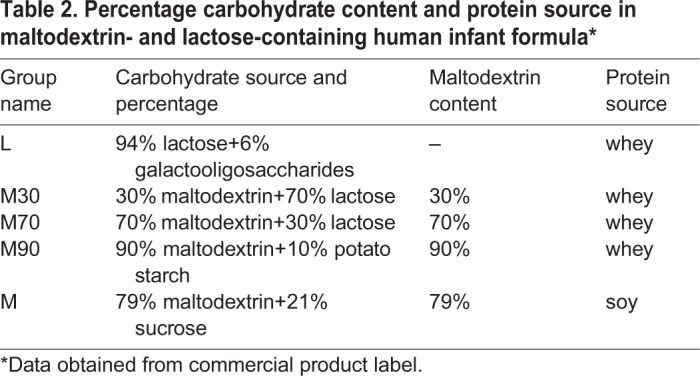
Percentage carbohydrate content and protein source in maltodextrin- and lactose-containing human infant formula*

### Maltodextrin-dominant infant formula-induced intestinal injury is age dependent

NEC in preterm infants appears to chronologically peak within a specific range in postmenstrual age (between 30 and 32 weeks postmenstrual age), suggesting that there is a biological developmental timeline to disease risk (Yee et al., 2012). To investigate whether maltodextrin-dominant formula feeding induced intestinal injury follows a biological developmental timeline, the maltodextrin-induced intestinal injury model was conducted in older, 9- to 10-day-old mice. Relative to a control dam-fed group (D), gross morphological changes of the small intestine were present at both time points but demonstrated increased involvement in the younger, 5- to 6-day-old mice (MH) compared to the older, 9- to 10-day-old mice (MH1) (27.4±12.1% versus 3.8±5.5%, respectively; *P*<0.001; Fig. S3). These data confirm the developmental vulnerability of intestinal injury in this model.

## DISCUSSION

NEC is defined by the culmination of several pathogenic features with unpredictable penetrance in preterm infants, owing to variable degrees of host vulnerability. These factors make it challenging to generate an all-encompassing animal model that mirrors the disease process in preterm infants. Several models have been developed to improve our current understanding of NEC pathogenesis and to identify potential therapeutic targets (Barlow et al., 1974; Tian et al., 2010). However, each animal model has unique advantages and disadvantages. Murine models offer many advantages, including the genetic similarity of inbred strains, the opportunity to utilize transgenic mouse models, short life cycle, high reproductive rates and lower cost. However, the current murine models of NEC do not focus on specific nutrient-processing vulnerabilities and do not have a high penetrance of the NEC phenotype. Thus, we aimed to establish an alternative mouse model that takes advantage of enteral feeding as a trigger of intestinal injury and is also highly robust and reproducible. The goal was to generate a nutrient-derived model of intestinal injury that would mimic the early stages of intestinal injury that may predispose to the genesis of NEC.

Table 3 summarizes the features of the maltodextrin-induced intestinal injury model. In this model, the collective markers of intestinal injury that encompassed many of the features of NEC were optimally represented in the maltodextrin plus hypoxia group relative to the control, dam-fed group. We observed a significant positive correlation between intestinal injury and percentage dilatation. It is possible that maltodextrin-containing formula feeding in mice results in altered bacterial metabolism, and, as a result of bacterial fermentation, gas is generated, leading to enhanced dilatation (Kien, 1990). Excessive gas production may lead to distension and affect vascular perfusion, leading to hypoxia and ischemia, which subsequently cause an NEC-like intestinal injury. In addition to gas production, intestinal distension may be a feature of poor motility with impairment of the gut-hormone axis or the neuroendocrine system. Interestingly, the addition of *K. pneumoniae* did not add any additional features to the model. Adding bacteria did increase small intestinal dilatation relative to maltodextrin alone and increased mortality, although the latter did not reach statistical significance. Interestingly, there was a suggestion that *Klebsiella* attenuated the increase in or protected against the overall level of intestinal injury, and attenuated the claudin-3 expression induced by the maltodextrin with hypoxia group, but these findings were also not statistically significant. The exposures in this model had differential effects on the markers of intestinal injury. Maltodextrin appeared to be the primary driver for morphological changes, whereas hypoxia exposure was the primary driver of MUC2 expression and modulating intestinal barrier proteins. Exposure to maltodextrin and hypoxia thus results in a highly reproducible intestinal injury in a neonatal mouse with intestinal dilatation, loss of villi, changes in intestinal barrier function, cell necrosis and increased intestinal cytokine production.

**Table 3.**
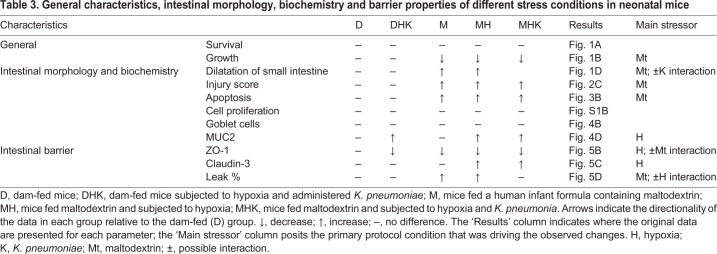
**General characteristics, intestinal morphology, biochemistry and barrier properties of different stress conditions in neonatal mice**

Absence of intestinal injury in the DHK group suggests that several essential factors present within breast milk could be important for maintaining epithelial health, gut barrier function and regulation of inflammation preventing intestinal injury (Dickinson et al., 1998; Gopalakrishna et al., 2019). However, the DHK group did demonstrate an increase in MUC2 and a decrease in ZO-1 expression relative to the dam-fed control group, supporting the role of hypoxia as a primary driver of these changes.

A well-regulated intestinal barrier function is needed to prevent intestinal translocation of luminal antigens that may lead to intestinal inflammation and injury. Disruption of intestinal barrier function leads to intestinal mucosal injury and inflammation in diseases such as inflammatory bowel disease and NEC (Westerbeek et al., 2006), as evidenced by enhanced intestinal permeability (Hollander, 1999; Moore et al., 2016; Turner, 2009). The mucus layer provides the first line of innate host defense, specifically due to MUC2, a secretory mucin produced by goblet cells. Goblet cells limit the interaction between pathogens and the intestinal epithelium by synthesizing MUC2 (Andrianifahanana et al., 2006; Hodzic et al., 2017; Mara et al., 2018). Altered MUC2 secretion has been implicated in animal NEC models as well as in inflammatory bowel disease (Clark et al., 2006; Tawiah et al., 2018b; Wu et al., 2019). Higher MUC2 expression was seen in groups DHK, MH and MHK, but not in group M, compared to control group D, confirming the correlation of hypoxia with MUC2 expression. Higher MUC2 expression may be related to endoplasmic reticulum stress within goblet cells, leading to dysfunctional intestinal barrier function (Tawiah et al., 2018a). Although the number of goblet cells was not different between groups, we did quantify differences in MUC2 expression. Meprin β, a proteolytic enzyme, is required to cleave and release MUC2 from goblet cells. There are published reports that meprin β expression is upregulated by bacteria (Schutte et al., 2014) but inhibited by a glycoprotein, fetuin-A (also known as AHSG), which has been found in breastmilk (Zhang et al., 2016). This may explain the increase in MUC2 expression with hypoxia and *Klebsiella* in the maltodextrin groups, but not dam-fed groups. Further studies will evaluate whether this enzyme is differentially upregulated in the formula groups with stressors (hypoxia and bacteria) relative to the dam-fed groups.

Tight junction integrity is regulated in both physiologic and pathologic states (Moore et al., 2016; Shen and Turner, 2006) to normally prevent intestinal inflammation (Sturm and Dignass, 2008). The integrity of tight junctions is pivotal to intestinal epithelial homeostasis and intestinal permeability. Previous studies in animal NEC models have shown changes in expression levels of claudin-3, occludin and ZO-1 (Clark et al., 2006; Khailova et al., 2009; Rentea et al., 2012; Shiou et al., 2011). Consistent with previous animal NEC studies (Clark et al., 2006; Rentea et al., 2012; Shi et al., 2018), we found a decrease in ZO-1 expression with an increase in claudin-3. Reduced expression of ZO-1 was evident in all groups, including DHK, where the mice did not show histological evidence of intestinal injury, suggesting that lower expression could be a predisposing factor and could partly explain changes in intestinal permeability in our intestinal injury model.

Inflammatory mediators have been implicated in the pathogenesis of NEC, with the resulting imbalance between pro-inflammatory and anti-inflammatory cytokines leading to disease progression and intestinal tissue damage (Cho et al., 2016; Markel et al., 2006; Martin and Walker, 2006; Neurath, 2014). Our cytokine results confirm an accompanied inflammatory response with the evidence of intestinal injury. In this model, the inflammatory response is evident early at day 1, with resolution by day 4, except for a persistent IL-6 response. The time course of the IL-6 response over several days is consistent with the literature defining cytokine responses with inflammation and injury (Loram et al., 2007). We did not identify inflammatory infiltrates or loss of villi on histological analysis at day 1, with the exception of vacuolated villus epithelial cells in experimental groups compared to the dam-fed group (Liu et al., 2009). The vacuolization of enterocytes could be due to alteration in endocytosis and/or exocytosis (Remis et al., 2014). Furthermore, enterocyte vacuolization may indicate reduced cell turnover and/or maturation, thus suggesting an impact on mucosal development (Yeruva et al., 2016). The finding of inflammatory changes prior to the development of morphological changes is a positive feature in this model as it potentially offers a window of opportunity to introduce preventive strategies in interrupting the cycle of nutrient-induced intestinal injury in hosts with immature digestive and metabolic capabilities.

The maltodextrin mouse model is consistent with the maltodextrin intestinal injury model in preterm piglets. Thymann et al. (2009) have shown that maltodextrin-containing formula fed to preterm piglets is sufficient to induce NEC, as evidenced by a significant loss in body weight, villus disruption, pneumatosis and inflammation. Compared to the piglet model of maltodextrin-induced intestinal injury, the neonatal mouse has the advantages of a shorter experimental duration, lower overall cost and the ability to utilize transgenic models to interrogate mechanisms.

NEC-like injury is observed within a short postnatal developmental window that correlates with the timing of the highest risk in premature infants (Yee et al., 2012). The maltodextrin-dominant formula-induced intestinal injury mouse model also demonstrated a differential risk based on postnatal age and thus provides a basis for understanding developmental vulnerability for intestinal damage. The impact of maltodextrin is likely to be multifactorial, with epithelial cell-driven mechanisms, but the carbohydrate likely also alters the microbial phenotype and gut inflammation (Laudisi et al., 2019; Nickerson et al., 2015). In addition to epithelium-dependent pathways, immature gut microcirculation and reduced postprandial hyperemia may be an important factor leading to intestinal injury in younger, immature mice versus older, more mature mice (Chen et al., 2019).

A limitation of our study is that the maturation of maltodextrin digestion in human infants is unknown; thus, the specific contribution of this nutrient to intestinal injury of the preterm infant remains speculative. Although maltodextrin formulas tend to be limited to term formulas with no adverse reports, maltodextrin is known to be included in infant-feeding thickeners, which have been linked to the development of NEC in preterm infants. A second limitation is that we used multiple prespecified, commercially available formulas. This limited our ability to match for every ingredient, and, thus, additive effects from other components could not be fully excluded. We do posit, however, that the observed increasing intestinal dilatation with increasing maltodextrin content, in conjunction with prior published studies using maltodextrin, non-soy formulas to induce intestinal injury in preterm piglets (Buddington et al., 2018; Thymann et al., 2009), support the hypothesis that maltodextrin is a primary driver of intestinal injury in our model as well. Overall, our study shows that maltodextrin-dominant human infant formula feeding in combination with hypoxia induces a highly reproducible model of small intestinal injury in the neonatal mouse.

## MATERIALS AND METHODS

### Statement of ethics

All animal experiments were carried out in accordance with the recommendations in the Guide for the Care and Use of Laboratory Animals of the National Institutes of Health. All procedures were approved by the Institutional Animal Care and Use Committee at the Beth Israel Deaconess Medical Center (BIDMC), Boston, MA, USA.

### Induction of intestinal injury in neonatal mice

C57BL/6 wild-type (WT) mice were purchased from The Jackson Laboratory (Bar Harbor, ME, USA). Breeding pairs of C57BL/6 WT mice were housed in the BIDMC animal facility with controlled humidity and temperature and standard light-dark cycles. Access to food and water was available *ad libitum*. Dams were allowed to deliver naturally, and 5- to 6-day-old pups with a body weight between 3.0 g and 4.0 g were used for the experiments. Mouse pups were placed in a double-walled plexiglass isolette (Air-Shields, Hatboro, PA, USA) maintained at 34°C during the course of the experiment. Preliminary experiments were conducted to determine the formula that resulted in the most prevalent and reproducible gross appearance of intestinal injury. Formulas tested included (1) a maltodextrin-dominant formula (79%) with soy protein (M), (2) predominantly lactose (no maltodextrin) and whey protein (L), (2) 30% maltodextrin and whey protein (M30), (3) 70% maltodextrin and whey protein (M70), (4) 90% maltodextrin and whey protein (M90), and (5) 79% maltodextrin and soy protein (M) (Table 2, Table S2). The latter formula, 79% maltodextrin and soy protein, was chosen for the remainder of the studies, given the highest prevalence of gross intestinal injury in the model when using this formula (Fig. 7).

The pups were divided into the following experimental groups: dam-fed only (D, served as the control group), D+hypoxia+*K. pneumoniae* (DHK), 79% maltodextrin+21% sucrose with soy protein containing human infant formula (M) only, M+hypoxia (MH) and M+hypoxia+*K. pneumoniae* (MHK).

All of the formulas were prepared according to the manufacturer's instructions. Mouse pups were formula fed using 50 μl/g body weight, five times/day from day 0 to day 4 by performing orogastric gavage using a 1.9F silastic catheter (Argon Medical Devices, Frisco, TX, USA; Fig. S4). In the hypoxia groups, mice were subjected to hypoxia (5% O_2_, 95% N_2_) for 10 min, twice a day (right after the first and last feeding) for 4 days. The oxygen levels were monitored continuously with a MAXO2+AE oxygen analyzer (Maxtec, West Valley City, UT, USA). For the *Klebsiella* groups, *K. pneumoniae* (ATCC 10031, Manassas, VA, USA) was grown to log phase using Difco™ nutrient broth medium (Becton Dickinson, Sparks, MD, USA). Bacteria were diluted in PBS, and the optical density at 600 nm (OD_600_) was measured. The culture was centrifuged at 9000 ***g*** for 30 min, and the pellet was resuspended in PBS. In the bacteria-fed groups, mice were gavaged with 2×10^7^ colony-forming units *K. pneumoniae*/g body weight five times a day in the DHK group and with each feeding in the MHK group.

To evaluate the effect of maltodextrin-containing formula on body weight, mouse pups were weighed every morning before the first feeding. On day 4 of the experiment, the mice were sacrificed, and the intestines were removed as a whole then photographed, and percentage dilatation of the small intestine measured as a percentage of total small intestinal length was quantified. Approximately 2.0 cm of distal ileum proximal to the cecum was then collected and fixed in 10% buffered formalin and embedded in paraffin, sectioned and stained with H&E using a standard protocol for histological examination. The same tissue section was also used for immunohistochemistry (IHC) and immunofluorescence (IF) staining. Any additional areas that appeared to be distended were saved at −80°C until further use. Grossly involved segments were used for histopathology and cytokine analysis.

### Intestinal injury severity assessment

Upon sacrifice, the large and small intestines were carefully removed and visually evaluated for signs of intestinal injury. Grossly, intestinal dilatation was the predominant feature and was quantified using ImageJ software and shown as percentage dilatation [(sum of the lengths of dilatated segments/total segment length)×100]. Small intestinal tissue sections stained with H&E were used for histological evaluation of the presence and/or degree of intestinal injury using the NEC histologic injury scoring system described by Caplan et al. (1994) with modifications. The severity of intestinal injury was graded by two independent observers using an intestinal tissue injury score (0-4). Histological changes were graded as follows: grade 0, intact villi with no damage; grade 1, epithelial cell lifting or separation; grade 2, partial loss of villi; grade 3, loss of entire villi; and grade 4, transmural necrosis. Tissues samples with histological scores of 2 or higher were considered positive for intestinal injury. A histological score was assigned to each specimen based on the area of the worst injury.

### Protein extraction and quantification

Intestinal tissues were homogenized, sonicated and the proteins were extracted using RIPA buffer [50 mM Tris-HCl (pH 8.0), 150 mM NaCl, 1% Nonidet P-40, 0.5% sodium deoxycholate, 0.1% SDS], 1 mM orthovanadate, complete protease inhibitor mixture (Roche Diagnostics, Indianapolis, IN, USA), and 1 mM PMSF on ice for 20 min. Lysates were centrifuged at 14,000 ***g*** for 20 min at 4°C. The resulting supernatants were collected, and protein concentrations were determined using the colorimetric Bradford reagent (Bio-Rad, Hercules, CA, USA). Tissue homogenates were stored at −80°C for subsequent analysis by Meso Scale Discovery (MSD; Kenilworth, NJ, USA) and ELISA.

### Measurement of cytokines in tissue lysates

Inflammatory cytokines were quantified from the dilated portions of the small intestine (except in the dam-fed groups where there was no injury) on day 1 and day 4 of the experiment. Tissue homogenate samples were analyzed using the MSD U-PLEX Plus Cytokine Panel (Mouse) Kit (IFN-γ, IL-17A, IL-1β, IL-23, IL-4, IL-6, KC/GRO and TNF-α). All assays were carried out according to the manufacturer's specifications. Quality and assay standard controls were included for independent runs as per the manufacturer's protocol. All MSD assays were read using a MESO QuickPlex SQ 120, and analysis was performed using DISCOVERY WORKBENCH 4.0 (MSD). Analyte values were reported in pg/mg protein.

### ZO-1 ELISA

The levels of ZO-1 in tissue homogenates were measured using an ELISA kit (LSBio, Seattle, WA, USA) according to the manufacturer's recommendations. Results were reported in pg/µg protein.

### TUNEL staining

TUNEL staining was performed using an *In-Situ* Cell Death Detection Kit, TMR Red (Roche Diagnostics, Indianapolis, IN, USA) according to the manufacturer's instructions. Approximately 2.0 cm of distal ileum sections proximal to colon was used. The percentage of TUNEL-positive-cell-containing crypts in more than 100 randomly chosen crypts was calculated for each slide.

### Goblet cells quantification

Ileal sections were stained with AB for the quantification of goblet cells, as described previously (Singh et al., 2019). For each animal, the number of goblet cells in the ileum was counted on at least 30 well-orientated villus-crypt units. Villus epithelial cells were counted from the entrance of the crypt opening to the beginning of the curve at the villus tip. Results were expressed as the number of goblet cells per 100 villus epithelial cells. Areas with full loss of villi were not included for analysis.

### Immunohistochemistry for Ki67

Ileal tissues were immunostained using a rabbit anti-Ki67 (Thermo Fisher Scientific, Waltham, MA, USA; RM-9106-S1) antibody followed by a biotinylated donkey anti-rabbit IgG (Vector Laboratories, Burlingame, CA, USA) and then by avidin-horseradish peroxidase conjugates (Vector Laboratories). Positive cells were visualized after incubation with 3,3′-diaminobenzidine (Sigma-Aldrich) for 2-5 min and counterstained with Hematoxylin. The total number of Ki67-positive cells was determined for 100 crypts each slide.

### Immunofluorescence studies

Immunofluorescence was performed with MUC2, claudin-3 and ZO-1 antibodies. Paraffin sections were deparaffinized and treated with heat-mediated antigen retrieval using sodium citrate buffer (pH 6.0). After three washes with TBS, the sections were incubated with 5% normal donkey serum (Jackson ImmunoResearch, West Grove, PA, USA) for 1 h at room temperature to block non-specific binding. Sections were then incubated with rabbit anti-MUC2 (1:200; H-300, Santa Cruz Biotechnology, sc-15534), rabbit anti-claudin 3 (1:100; Invitrogen, 34-1700) and rabbit anti-ZO-1 (1:250; Invitrogen, 61-7300) overnight at 4°C. The slides were washed three times and incubated with Cy3-conjugated donkey anti-rabbit secondary antibody (1:300; Jackson ImmunoResearch). Samples were counterstained with Hoechst 33342 (Invitrogen) and washed three times with TBS. The slides were mounted with Prolong Gold anti-fade mounting medium (Invitrogen). Nuclei were counterstained with diamidino-2 phenylindole (DAPI) (Sigma-Aldrich, St Louis, MO, USA). The slides were imaged using a fluorescent microscope (Nikon, Japan), and intensity was calculated using ImageJ software.

### Measurement of intestinal permeability

Intestinal permeability assay was performed as described previously (Singh et al., 2019). Briefly, mice were gavaged with FITC-dextran 4000 (FD-4) (Sigma-Aldrich) in PBS at a concentration of 600 mg/kg body weight. Blood samples were collected after 3.5 h and diluted 1:100 in PBS to measure the fluorescence intensity using a fluorospectrophotometer (Hitachi F-2000, Tokyo, Japan) with an excitation wavelength of 480 nm and an emission wavelength of 520 nm. The concentration of FD-4 in serum samples was calculated using a known standard of serially diluted FD-4. Serum samples of mice that did not receive any FD-4 were used to determine the background, and the results were expressed as percentage leak.

### Contribution of formula type and postnatal age to intestinal injury

To further interrogate the impact of maltodextrin and other potential compositional factors in formula, additional formula groups were studied across a range of carbohydrate sources. These additional formula groups included lactose with whey (L), 30% maltodextrin with whey (M30), 70% maltodextrin and whey (M70), and 90% maltodextrin and whey (M90). The degree of intestinal dilatation, a hallmark feature of this intestinal injury model, was quantified for each group. Finally, a group of older mice (9-10 postnatal days versus 5-6 postnatal days) was evaluated to assess whether the model was developmentally regulated.

### Statistical analysis

All data were evaluated for normality using the Shapiro test statistic. Data were summarized as median and IQR. Kaplan–Meier curves were reported, and survival analysis was tested by log-rank test. Body weight data were analyzed by two-way ANOVA after rank normal transformation with Tukey's post hoc analysis to adjust for multiple comparisons. Mann–Whitney test was used when comparing two groups, and Kruskal–Wallis test was used when performing comparisons across multiple groups, followed by Dunn's pairwise comparison to adjust for multiple comparisons. To determine the correlation between percentage dilatation and intestinal injury, Spearman's correlation was used. All data analyses were performed using R software (version 3.6.0, R Core Team, Vienna, Austria, 2018) within R Studio interface using tidyverse, dplyr, survival, FSA, dunn.test, rcompanion and DescTools packages. *P*<0.05 was considered statistically significant.

## Supplementary Material

Supplementary information

## References

[DMM044776C1] Andrianifahanana M., Moniaux N. and Batra S. K. (2006). Regulation of mucin expression: mechanistic aspects and implications for cancer and inflammatory diseases. *Biochim. Biophys. Acta Rev. Cancer* 1765, 189-222. 10.1016/j.bbcan.2006.01.002 16487661

[DMM044776C2] Barlow B., Santulli T. V., Heird W. C., Pitt J., Blanc W. A. and Schullinger J. N. (1974). An experimental study of acute neonatal enterocolitis—the importance of breast milk. *J. Pediatr. Surg.* 9, 587-595. 10.1016/0022-3468(74)90093-1 4138917

[DMM044776C3] Buddington R. K., Davis S. L. and Buddington K. K. (2018). The risk of necrotizing enterocolitis differs among preterm pigs fed formulas with either lactose or maltodextrin. *J. Pediatr. Gastroenterol. Nutr.* 66, e61-e66. 10.1097/MPG.0000000000001707 28806296

[DMM044776C4] Caplan M. S., Hedlund E., Adler L. and Hsueh W. (1994). Role of asphyxia and feeding in a neonatal rat model of necrotizing enterocolitis. *Pediatr. Pathol.* 14, 1017-1028. 10.3109/15513819409037698 7855004

[DMM044776C5] Chen Y., Koike Y., Chi L., Ahmed A., Miyake H., Li B., Lee C., Delgado-Olguin P. and Pierro A. (2019). Formula feeding and immature gut microcirculation promote intestinal hypoxia, leading to necrotizing enterocolitis. *Dis. Model. Mech.* 12, dmm040998 10.1242/dmm.040998 31704804 PMC6918740

[DMM044776C6] Cho S. X., Berger P. J., Nold-Petry C. A. and Nold M. F. (2016). The immunological landscape in necrotising enterocolitis. *Expert Rev. Mol. Med.* 18, 125-117. 10.1017/erm.2016.13 PMC5001507 27341512

[DMM044776C7] Clark J. A., Doelle S. M., Halpern M. D., Saunders T. A., Holubec H., Dvorak K., Boitano S. A. and Dvorak B. (2006). Intestinal barrier failure during experimental necrotizing enterocolitis: protective effect of EGF treatment. *Am. J. Physiol. Gastrointest. Liver Physiol.* 291, G938-G949. 10.1152/ajpgi.00090.2006 16798726

[DMM044776C8] Dickinson E. C., Gorga J. C., Garrett M., Tuncer R., Boyle P., Watkins S. C., Alber S. M., Parizhskaya M., Trucco M., Rowe M. I. et al. (1998). Immunoglobulin A supplementation abrogates bacterial translocation and preserves the architecture of the intestinal epithelium. *Surgery* 124, 284-290. 10.1016/S0039-6060(98)70132-1 9706150

[DMM044776C9] Gopalakrishna K. P., Macadangdang B. R., Rogers M. B., Tometich J. T., Firek B. A., Baker R., Ji J., Burr A. H. P., Ma C., Good M. et al. (2019). Maternal IgA protects against the development of necrotizing enterocolitis in preterm infants. *Nat. Med.* 25, 1110-1115. 10.1038/s41591-019-0480-9 31209335 PMC7424541

[DMM044776C10] Hodzic Z., Bolock A. M. and Good M. (2017). The role of mucosal immunity in the pathogenesis of necrotizing enterocolitis. *Front. Pediatr.* 5, e137-17 10.3389/fped.2017.00040 PMC5334327 28316967

[DMM044776C11] Hollander D. (1999). Intestinal permeability, leaky gut, and intestinal disorders. *Curr. Gastroenterol. Rep.* 1, 410-416. 10.1007/s11894-999-0023-5 10980980

[DMM044776C12] Howles P. N., Stemmerman G. N., Fenoglio-Preiser C. M. and Hui D. Y. (1999). Carboxyl ester lipase activity in milk prevents fat-derived intestinal injury in neonatal mice. *Am. J. Physiol.* 277, G653-G661. 10.1152/ajpgi.1999.277.3.G653 10484391 PMC2583025

[DMM044776C13] Isani M. A., Delaplain P. T., Grishin A. and Ford H. R. (2018). Evolving understanding of neonatal necrotizing enterocolitis. *Curr. Opin. Pediatr.* 30, 417-423. 10.1097/MOP.0000000000000629 29601338

[DMM044776C14] Khailova L., Dvorak K., Arganbright K. M., Halpern M. D., Kinouchi T., Yajima M. and Dvorak B. (2009). Bifidobacterium bifidum improves intestinal integrity in a rat model of necrotizing enterocolitis. *Am. J. Physiol. Gastrointest. Liver Physiol.* 297, G940-G949. 10.1152/ajpgi.00141.2009 20501441 PMC2777452

[DMM044776C15] Kien C. L. (1990). Colonic fermentation of carbohydrate in the premature infant: possible relevance to necrotizing enterocolitis. *J. Pediatr.* 117, S52-S58. 10.1016/S0022-3476(05)81131-X 2194012

[DMM044776C16] Kwok T. C., Dorling J. and Gale C. (2019). Early enteral feeding in preterm infants. *Semin. Perinatol.* 43, 151159-151111. 10.1053/j.semperi.2019.06.007 31443906

[DMM044776C17] Laudisi F., Di Fusco D., Dinallo V., Stolfi C., Di Grazia A., Marafini I., Colantoni A., Ortenzi A., Alteri C., Guerrieri F. et al. (2019). The food additive maltodextrin promotes endoplasmic reticulum stress-driven mucus depletion and exacerbates intestinal inflammation. *Cell Mol. Gastroenterol. Hepatol.* 7, 457-473. 10.1016/j.jcmgh.2018.09.002 30765332 PMC6369223

[DMM044776C18] Liu Y., Zhu L., Fatheree N. Y., Liu X., Pacheco S. E., Tatevian N. and Rhoads J. M. (2009). Changes in intestinal Toll-like receptors and cytokines precede histological injury in a rat model of necrotizing enterocolitis. *Am. J. Physiol. Gastrointest. Liver Physiol.* 297, G442-G450. 10.1152/ajpgi.00182.2009 19608731 PMC2739826

[DMM044776C19] Loram L. C., Themistocleous A. C., Fick L. G. and Kamerman P. R. (2007). The time course of inflammatory cytokine secretion in a rat model of postoperative pain does not coincide with the onset of mechanical hyperalgesia. *Can. J. Physiol. Pharmacol.* 85, 613-620. 10.1139/Y07-054 17823623

[DMM044776C20] Mara M. A., Good M. and Weitkamp J.-H. (2018). Innate and adaptive immunity in necrotizing enterocolitis. *Semin. Fetal. Neonatal. Med.* 23, 394-399. 10.1016/j.siny.2018.08.002 30146477 PMC6269198

[DMM044776C21] Markel T. A., Crisostomo P. R., Wairiuko G. M., Pitcher J., Tsai B. M. and Meldrum D. R. (2006). Cytokines in necrotizing enterocolitis. *Shock* 25, 329-337. 10.1097/01.shk.0000192126.33823.87 16670633

[DMM044776C22] Martin C. R. and Walker W. A. (2006). Intestinal immune defences and the inflammatory response in necrotising enterocolitis. *Semin. Fetal. Neonatal. Med.* 11, 369-377. 10.1016/j.siny.2006.03.002 16690363

[DMM044776C23] Moore S. A., Nighot P., Reyes C., Rawat M., McKee J., Lemon D., Hanson J. and Ma T. Y. (2016). Intestinal barrier dysfunction in human necrotizing enterocolitis. *J. Pediatr. Surg.* 51, 1907-1913. 10.1016/j.jpedsurg.2016.09.011 27720222 PMC5245981

[DMM044776C24] Nanthakumar N. N., Fusunyan R. D., Sanderson I. and Walker W. A. (2000). Inflammation in the developing human intestine: a possible pathophysiologic contribution tonecrotizing enterocolitis. *Proc. Natl Acad. Sci. USA* 97, 6043-6048. 10.1073/pnas.97.11.6043 10823949 PMC18555

[DMM044776C25] Neu J. and Walker W. A. (2011). Necrotizing enterocolitis. *N. Engl. J. Med.* 364, 255-264. 10.1056/NEJMra1005408 21247316 PMC3628622

[DMM044776C26] Neurath M. F. (2014). Cytokines in inflammatory bowel disease. *Nat. Rev. Immunol.* 14, 329-342. 10.1038/nri3661 24751956

[DMM044776C27] Nickerson K. P., Chanin R. and McDonald C. (2015). Deregulation of intestinal anti-microbial defense by the dietary additive, maltodextrin. *Gut Microbes* 6, 78-83. 10.1080/19490976.2015.1005477 25738413 PMC4615306

[DMM044776C28] Niño D. F., Sodhi C. P. and Hackam D. J. (2016). Necrotizing enterocolitis: new insights into pathogenesis and mechanisms. *Nat. Rev. Gastroenterol. Hepatol.* 13, 590-600. 10.1038/nrgastro.2016.119 27534694 PMC5124124

[DMM044776C29] Remis N. N., Wiwatpanit T., Castiglioni A. J., Flores E. N., Cantú J. A. and García-Añoveros J. (2014). Mucolipin co-deficiency causes accelerated endolysosomal vacuolation of enterocytes and failure-to-thrive from birth to weaning. *PLoS Genet.* 10, e1004833-e19. 10.1371/journal.pgen.1004833 25521295 PMC4270466

[DMM044776C30] Rentea R. M., Liedel J. L., Welak S. R., Cassidy L. D., Mayer A. N., Pritchard K. A. Jr., Oldham K. T. and Gourlay D. M. (2012). Intestinal alkaline phosphatase administration in newborns is protective of gut barrier function in a neonatal necrotizing enterocolitis rat model. *J. Pediatr. Surg.* 47, 1135-1142. 10.1016/j.jpedsurg.2012.03.018 22703783

[DMM044776C31] Rich B. S. and Dolgin S. E. (2017). Necrotizing enterocolitis. *Pediatr. Rev.* 38, 552-559. 10.1542/pir.2017-0002 29196510

[DMM044776C32] Schutte A., Ermund A., Becker-Pauly C., Johansson M. E. V., Rodriguez-Pineiro A. M., Backhed F., Muller S., Lottaz D., Bond J. S. and Hansson G. C. (2014). Microbial-induced meprin beta cleavage in MUC2 mucin and a functional CFTR channel are required to release anchored small intestinal mucus. *Proc. Natl. Acad. Sci. USA* 111, 12396-12401. 10.1073/pnas.1407597111 25114233 PMC4151749

[DMM044776C33] Shen L. and Turner J. R. (2006). Role of epithelial cells in initiation and propagation of intestinal inflammation. Eliminating the static: tight junction dynamics exposed. *Am. J. Physiol. Gastrointest. Liver Physiol.* 290, G577-G582. 10.1152/ajpgi.00439.2005 16537969

[DMM044776C34] Shi Y., Liu T., Zhao X., Yao L., Hou A., Fu J. and Xue X. (2018). Vitamin D ameliorates neonatal necrotizing enterocolitis via suppressing TLR4 in a murine model. *Pediatr. Res.* 83, 1024-1030. 10.1038/pr.2017.329 29281615

[DMM044776C35] Shiou S.-R., Yu Y., Chen S., Ciancio M. J., Petrof E. O., Sun J. and Claud E. C. (2011). Erythropoietin protects intestinal epithelial barrier function and lowers the incidence of experimental neonatal necrotizing enterocolitis. *J. Biol. Chem.* 286, 12123-12132. 10.1074/jbc.M110.154625 21262973 PMC3069416

[DMM044776C36] Shulhan J., Dicken B., Hartling L. and Larsen B. M. K. (2017). Current knowledge of necrotizing enterocolitis in preterm infants and the impact of different types of enteral nutrition products. *Adv. Nutr. Int. Rev. J.* 8, 80-91. 10.3945/an.116.013193 PMC5227976 28096129

[DMM044776C37] Singh P., Ochoa-Allemant P., Brown J., Perides G., Freedman S. D. and Martin C. R. (2019). Effect of polyunsaturated fatty acids on postnatal ileum development using the fat-1 transgenic mouse model. *Pediatr. Res.* 85, 556-565. 10.1038/s41390-019-0284-0 30653193 PMC6397682

[DMM044776C38] Sodhi C. P., Fulton W. B., Good M., Vurma M., Das T., Lai C.-S., Jia H., Yamaguchi Y., Lu P., Prindle T. et al. (2018). Fat composition in infant formula contributes to the severity of necrotising enterocolitis. *Br. J. Nutr.* 120, 665-680. 10.1017/S0007114518001836 30176959 PMC6126914

[DMM044776C39] Sturm A. and Dignass A. U. (2008). Epithelial restitution and wound healing in inflammatory bowel disease. *World J. Gastroenterol.* 14, 348-353. 10.3748/wjg.14.348 18200658 PMC2679124

[DMM044776C40] Tawiah A., Cornick S., Moreau F., Gorman H., Kumar M., Tiwari S. and Chadee K. (2018a). High MUC2 mucin expression and misfolding induce cellular stress, reactive oxygen production, and apoptosis in goblet cells. *Am. J. Pathol.* 188, 1354-1373. 10.1016/j.ajpath.2018.02.007 29545196

[DMM044776C41] Tawiah A., Moreau F., Kumar M., Tiwari S., Falguera J. and Chadee K. (2018b). High MUC2 Mucin biosynthesis in goblet cells impedes restitution and wound healing by elevating endoplasmic reticulum stress and altered production of growth factors. *Am. J. Pathol.* 188, 2025-2041. 10.1016/j.ajpath.2018.05.013 29935164

[DMM044776C42] Thymann T., Møller H. K., Stoll B., Støy A. C. F., Buddington R. K., Bering S. B., Jensen B. B., Olutoye O. O., Siggers R. H., Mølbak L. et al. (2009). Carbohydrate maldigestion induces necrotizing enterocolitis in preterm pigs. *Am. J. Physiol. Gastrointest. Liver Physiol.* 297, G1115-G1125. 10.1152/ajpgi.00261.2009 19808655 PMC2850085

[DMM044776C43] Tian R., Liu S. X., Williams C., Soltau T. D., Dimmitt R., Zheng X. and De Plaen I. G. (2010). Characterization of a necrotizing enterocolitis model in newborn mice. *Int. J. Clin. Exp. Med.* 3, 293-302. 21072263 PMC2971546

[DMM044776C44] Turner J. R. (2009). Intestinal mucosal barrier function in health and disease. *Nat. Rev. Immunol.* 9, 799-809. 10.1038/nri2653 19855405

[DMM044776C45] Westerbeek E. A. M., van den Berg A., Lafeber H. N., Knol J., Fetter W. P. F. and van Elburg R. M. (2006). The intestinal bacterial colonisation in preterm infants: a review of the literature. *Clin. Nutr.* 25, 361-368. 10.1016/j.clnu.2006.03.002 16677741

[DMM044776C46] Wu R. Y., Li B., Koike Y., Määttänen P., Miyake H., Cadete M., Johnson-Henry K. C., Botts S. R., Lee C., Abrahamsson T. R. et al. (2019). Human milk oligosaccharides increase mucin expression in experimental necrotizing enterocolitis. *Mol. Nutr. Food Res.* 63, e1800658 10.1002/mnfr.201800658 30407734

[DMM044776C47] Yee W. H., Soraisham A. S., Shah V. S., Aziz K., Yoon W., Lee S. K. and the Canadian Neonatal Network (2012). Incidence and timing of presentation of necrotizing enterocolitis in preterm infants. *Pediatrics* 129, e298-e304. 10.1542/peds.2011-2022 22271701

[DMM044776C48] Yeruva L., Spencer N. E., Saraf M. K., Hennings L., Bowlin A. K., Cleves M. A., Mercer K., Chintapalli S. V., Shankar K., Rank R. G. et al. (2016). Formula diet alters small intestine morphology, microbial abundance and reduces VE-cadherin and IL-10 expression in neonatal porcine model. *BMC Gastroenterol.* 16, 40 10.1186/s12876-016-0456-x 27005303 PMC4804644

[DMM044776C49] Zani A. and Pierro A. (2015). Necrotizing enterocolitis: controversies and challenges. *F1000Res* 4, 1373-1310. 10.12688/f1000research.6888.1 PMC4753995 26918125

[DMM044776C50] Zhang L., van Dijk A. D. J. and Hettinga K. (2016). An interactomics overview of the human and bovine milk proteome over lactation. *Proteome Sci.* 15, 1 10.1186/s12953-016-0110-0 28149201 PMC5267443

