# Supplementary material for: Maltodextrin-induced intestinal injury in a neonatal mouse model
Source: Dis Model Mech. 2020 Aug 27;13(8):dmm044776. doi: 10.1242/dmm.044776 (PMC7473650; doi:10.1242/dmm.044776)
Supplement: Supplementary information [file dmm-13-044776-s1.pdf]

Table S1. Fold-change in cytokine expression in the maltodextrin-hypoxia (MH) group relative to the control, dam fed (D) group at day 1 and at day 4 of the intestinal injury model.

Cytokines	Day 1 MH/D	*P*	Day 4 MH/D	*P*	Day 1 to Day 4 description of temporal change
IFNγ	-3.0	0.01	+2.6	ns	Acute decrease with resolution
IL-17A	+883	<0.001	+1.0	ns	Acute increase with resolution
IL-1β	+3.5	<0.001	-0.8	ns	Acute increase with resolution
KC/GRO	+14.0	<0.001	+1.5	ns	Acute increase with resolution
IL-4	+1.1	ns	-0.2	<0.01	Acute increase with resolution
TNF-α	+15.3	<0.001	-0.6	<0.01	Acute increase with resolution
IL-23	+1.5	ns	+1.1	ns	No change
IL-6	+10.5	0.02	+20.0	<0.01	Persistent elevation

Data shown as mean fold change in the MH group relative to the D group. Wilcoxon rank sum tests were performed comparing expression levels of the MH group relative to the D group at Day 1 and at Day 4. Statistical significance threshold set at $P<0.05$. Description of temporal change from day 1 to day 4 was assigned as follows: "acute decrease with resolution" if a negative fold change difference existed at day 1 but not at day 4; acute increase with resolution" if a positive fold change difference existed at day 1 but not at day 4; "no change" if both day 1 and day 4 fold changes revealed no significant differences; and, "persistent elevation" if a positive fold change existed at both day 1 and day 4. Number of animals per group was n=10 for D and MH on day 1 and n=16 for D and MH on day 4. D = dam fed; MH = mice fed maltodextrin and subjected to hypoxia.

Table S2. Nutritional composition of Maltodextrin and Lactose containing human infant formula (per 100 cal)[1].

Nutrients	Lactose-dominant Infant formula (L)	Maltodextrin-containing Infant formula (M30)	Maltodextrin-containing Infant formula (M70)	Maltodextrin-containing Infant formula (M90)	Maltodextrin-containing Infant formula (M)[2]
Volume, ml	150	148	148	148	148
Protein, g	2.1	2.2	2.2	2.6	2.5
Protein source	whey	whey	whey	whey	soy
Fat, g	5.6	5.1	5.1	5.1	5.1
Fat source	high oleic safflower oil, soy oil, coconut oil	palm olein oil, soy oil, coconut oil, high oleic safflower or sunflower oil, single cell oil	palm olein oil, soy oil, coconut oil, high oleic safflower or sunflower oil, single cell oil	MCT oil, soy oil, high oleic sunflower oil, high 2-palmitic vegetable oil, CITREM, single cell oil	palm olein oil, soy oil, coconut oil, high oleic acid, soy oil, coconut oil, palm oil, sunflower oil, single cell oil
Carbohydrate, g	10.7	11.2	11.2	10.9	11.1
Carbohydrate source	94% lactose, 6% galactooligosaccharides	70% lactose, 30% maltodextrin	30% lactose, 70% maltodextrin	10% potato starch, 90% maltodextrin	79% maltodextrin, 21 % sucrose
Water, g	141	134	131	133	134
Linoleic Acid, mg	1000	900	900	777	920
Vitamin A, IU	300	300	300	300	300
Vitamin D, IU	75	75	60	45	60
Vitamin E, IU	1.5	2.0	2.0	2.0	2.0
Vitamin K, mcg	8	8	8	10	9
Thiamin (Vitamin B1), µg	100	100	100	73	80
Riboflavin (Vitamin B2), µg	160	140	140	152	94

Disease Models & Mechanisms • Supplementary information

Vitamin B6, µg	63	75	75	79	60
Vitamin B12, µg	0.26	0.30	0.30	0.30	0.33
Niacin, µg	1100	1050	1050	1028	1050
Folic acid, µg	16	15	15	15	16
Pantothenic acid, µg	470	450	450	730	500
Biotin, µg	4.6	4.4	4.4	2.0	5.0
Vitamin C, mg	9	10	10	15	12
Choline, µg	24	24	24	24	24
Inositol, mg	4.9	6	6	20	6
Calcium, mg	82	67	72	90	105
Phosphorus, mg	44	38	40	63	63
Magnesium, mg	6	7	7	9	11
Iron, mg	1.9	1.5	1.5	1.8	1.8
Zinc, mg	0.79	0.8	0.8	1.0	0.9
Manganese, µg	5	15	15	7	25
Copper, µg	95	80	80	98	80
Iodine, µg	6	12	12	15	15
Selenium, µg	2	3	3	2	3
Sodium, mg	25	27	27	38	40
Potassium, mg	110	108	108	98	116
Chloride, mg	68	65	65	79	70
Osmolality, mOsm/kg water	310	250	195	220	180

[1]Data was obtained from commercial product label.
[2]Main study diet

Figure S1

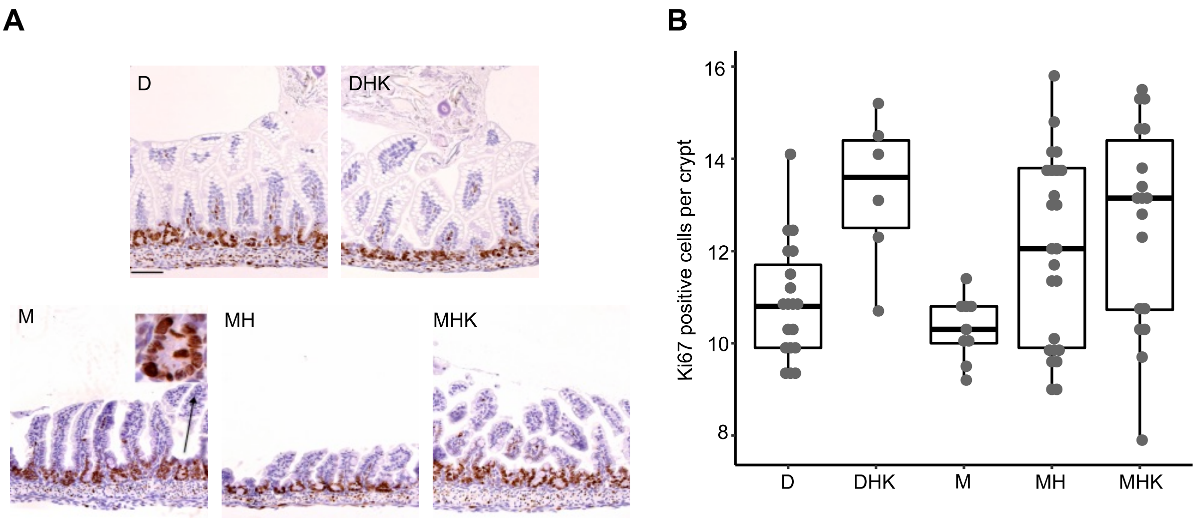

Fig. S1. Effect of maltodextrin containing formula on crypt cell proliferation. A) Representative images (200x, scale bar: 50 µm) of ileal sections immunostained for the proliferation marker Ki67. B) Quantification of Ki67 positive cells per crypt presented as a box dot plots showing median and IQR. D = dam fed, n=19; DHK = dam fed mice administered *Klebsiella pneumoniae* (K) by oral gavage and subjected to hypoxia (H), n=6; M = mice fed a human infant formula containing maltodextrin as a major carbohydrate source alone, n=9; MH = mice fed maltodextrin containing infant formula and subjected to hypoxia, n=24; MHK = mice fed maltodextrin containing infant formula along with *Klebsiella pneumoniae* and subjected to hypoxia, n=18.

Disease Models & Mechanisms • Supplementary information

Figure S2

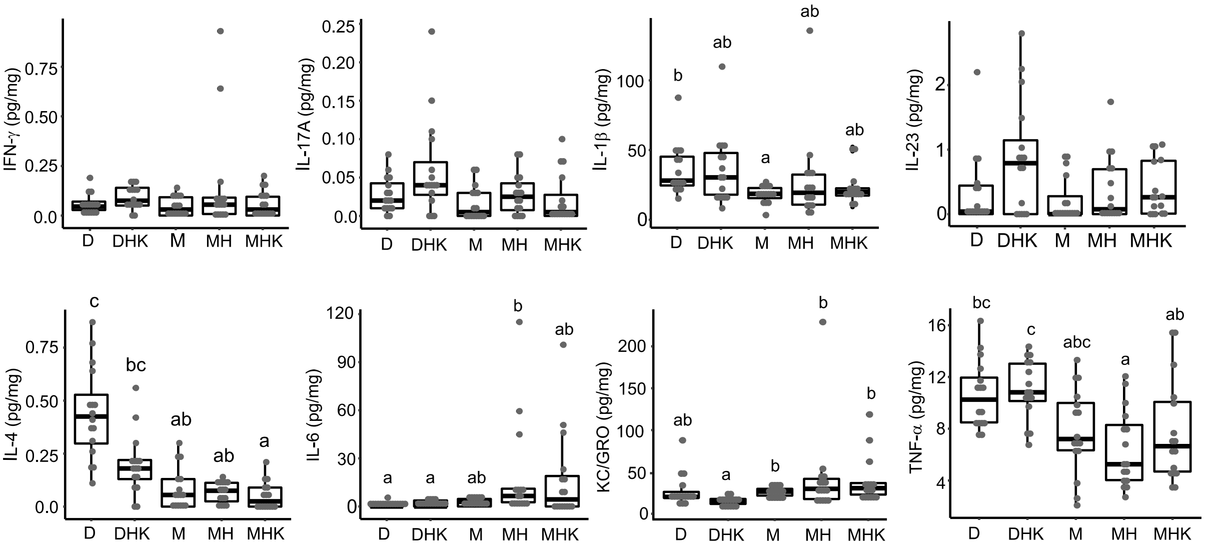

Fig. S2. Effect of maltodextrin containing formula feeding on intestinal cytokine production on day 4. Cytokines (IFN-γ, IL-17A, IL-1β, IL-23, IL-4, IL-6, KC/GRO and TNF-α) levels are presented as a box dot plots showing median and IQR. Labeled points without a common letter represent statistically significant differences, P<0.05. n=16 mice used in each group, D = dam fed; DHK = dam fed administered *Klebsiella pneumoniae* (K) by oral gavage and subjected to hypoxia (H); M = mice fed a human formula containing maltodextrin as a major carbohydrate source alone, n=9; MH = mice fed maltodextrin infant formula and subjected to hypoxia; MHK = mice fed maltodextrin containing infant formula along with *Klebsiella pneumoniae* and subjected to hypoxia.

Disease Models & Mechanisms • Supplementary information

Figure S3

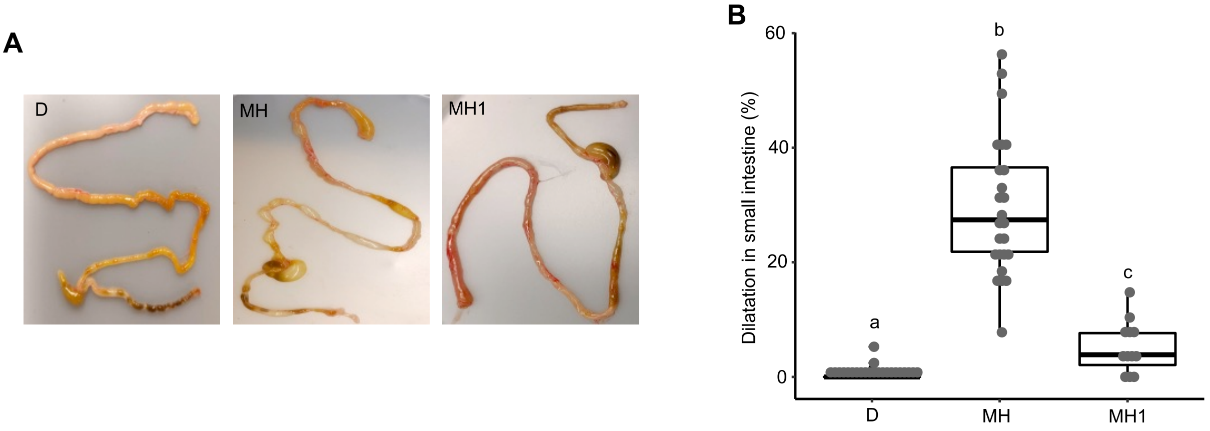

Fig. S3. Maltodextrin containing formula feeding results in increased dilatation in the small intestine in young mice (5-6 day old, MH) compared to controls and older mice (9-10 day old, MH1). A) Representative images of gross morphological changes in the small and large intestine from groups D, MH and MH1. B) Percent dilatation in the small intestine presented as box dot plots of median and IQR. Labeled points without a common letter represents statistically significant differences, $P<0.05$, D = 5-6 day old mice dam fed, n=23; MH = 5-6 day old mice fed maltodextrin containing infant formula and subjected to hypoxia, n=25; MH1 = 9-10 day old mice fed maltodextrin containing infant formula and subjected to hypoxia, n=12.

Disease Models & Mechanisms • Supplementary information

Figure S4

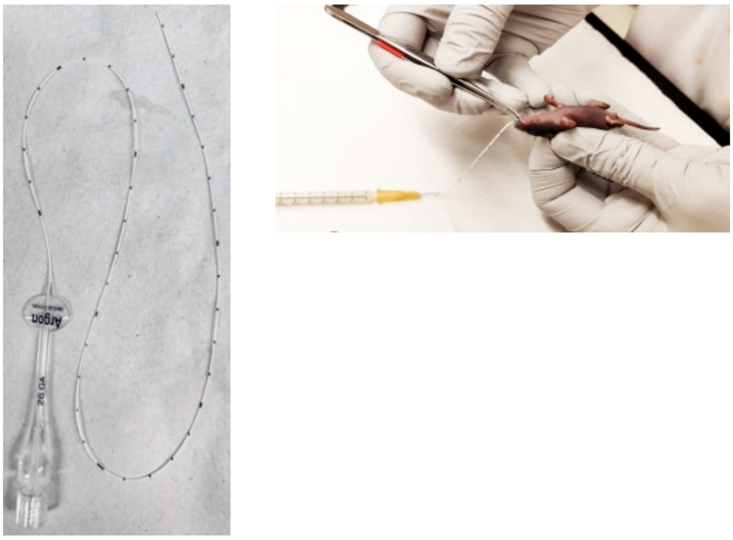

Fig. S4. Catheter-based feeding for oral gavage. Oral gavage is performed with a peripherally inserted central catheter (PICC line, 1.9F silastic catheter, Argon Medical Devices, Frisco, Texas) inserted to a depth of 2.0 cm in mouse pups.
